# Vitamin D as a Possible COVID-19 Prevention Strategy

**DOI:** 10.3390/ijms231810532

**Published:** 2022-09-11

**Authors:** Marie Bičíková, Ludmila Máčová, Martin Hill

**Affiliations:** Department of Steroids and Proteofactors, Institute of Endocrinology, Národni 8, 11694 Prague, Czech Republic

**Keywords:** vitamin D, COVID-19, LC-MS/MS determination

## Abstract

Vitamin D is no longer considered an agent only affecting calcium phosphate metabolism. A number of studies over the past few years have demonstrated its role in immunomodulation and its influence on the development and functioning of the brain and nervous system. In the current epidemiological crisis caused by coronavirus disease 2019 (COVID-19), the immunoprotective role of vitamin D has been discussed by some authors regarding whether it contributes to protection against this serious disease or whether its use does not play a role. Non-standard approaches taken by laboratories in examining the serum levels of the vitamin D metabolite calcidiol have contributed to inconsistent results. We examined the serum of 60 volunteers in the spring and autumn of 2021 who declared whether they were taking vitamin D at the time of sampling. Furthermore, the tested participants noted whether they had experienced COVID-19. A newly developed liquid chromatography–tandem mass spectrometry (LC-MS/MS) method was used to measure calcidiol levels. The analysis of variance (ANOVA) model of Statgraphics Centurion 18 statistical software from Statgraphics Technologies was used for calculations. The results of this study showed that those who took vitamin D suffered significantly less often from COVID-19 than those who did not take vitamin D.

## 1. Introduction

In addition to active movement, improvement of one’s nutrition and general lifestyle is a key feature in preventing serious diseases on one’s own. One such accessible tool may be micronutrients and vitamin D. For the past decade, reports have shown that, in addition to skeletal effects, vitamin D also has several protective effects in various areas of neurology, immunity, and oncology [1]. This is encouraging in the current economic climate and aging population as fewer funds will be available for health care. Until the age of about 60, people produce vitamin D themselves by irradiating the beta component of UV sunlight. The level of vitamin D produced in this natural way is sufficient to satisfy the basic needs of bone metabolism. Nowadays, with a lack of exercise in fresh air, vitamin D3 (cholecalciferol) supplementation is necessary, especially in winter. Year-round supplementation is required for people (especially women with a higher risk of osteoporosis) over 60 years of age.

The body’s supply of vitamin D is assessed by measuring the serum level of calcidiol (25-hydroxyvitamin D), which is its storage metabolite [2]. The active metabolite of cholecalciferol (calcitriol, 1,25-dihydroxyvitamin D) is classified as a neurosteroid with the ability to alter neuronal excitability. This fact also results in a number of beneficial effects [3]. The so-called extraskeletal effects of vitamin D are currently being discussed. Many psychiatric diseases have been correlated with low plasma levels of calcidiol [4,5], leading to speculation that sufficient vitamin D levels can prevent and even treat or at least alleviate some psychiatric disorders [6]. Vitamin D supplementation has been shown to be more effective in conjunction with active exercise. However, the results are contradictory. One of the reasons for this is the personalized response to vitamin D, as observed in high, mild, and low responders.

The positive and especially preventive effect of vitamin D on some cancer illnesses is also promising [7]. In combination with calcium, vitamin D has been recommended in recent years as an inexpensive, low-risk, and accessible approach to the treatment of hitherto difficult premenstrual syndrome (PMS). The combination, together with micronutrients, is able to eliminate or reduce the symptoms of premenstrual syndrome [8], menstrual disorders, and even endometriosis [9]. However, further studies on the heterogeneity of current measurements are needed to confirm the latter statement [10,11].

Vitamin D can play a positive role in such a serious disease due to its ability to regulate inflammatory processes. The anti-inflammatory effect of the active form of vitamin D (calcitriol) is explained by the ability of vitamin D to trigger regulatory cascades in immune cells, such as dendritic cells, monocytes/macrophages, and T and B cells [12]. The immunological effect of vitamin D lies in both congenital and acquired immunity [13]. Vitamin D is thus used to suppress autoimmunity and allergic reactions. Sufficient levels of vitamin D suppress inflammation and, additionally, promote antimicrobial innate immunity. During the coronavirus pandemic, interest in the protective effect of vitamin D (i.e., its active metabolite) in severe acute respiratory syndrome coronavirus 2 (SARS-CoV-2) infection has increased.

Hundreds of publications have appeared since 2020 on the relationship between vitamin D and coronavirus disease 2019 (COVID-19). Studies mostly note that vitamin D deficiency/insufficiency increases the odds of developing COVID-19 and that calcidiol concentrations were lower in patients than in controls; calcidiol levels were also lower in patients with severe COVID-19 compared to those of non-severe-COVID-19 patients [14,15]. However, the data are highly inconsistent. Some meta-analyses showed that vitamin D only reduces the severity of COVID-19 [16], while another study did not support a role for vitamin D in the course and outcome of COVID-19 at all [17].

At the peak of COVID-19 pandemic, healthcare staffs were exposed not only to enormous stress, but also to the daily risk of infection. In this work, we used generally accepted “gold-standard” liquid chromatography with mass spectrometry detection (LC-MS/MS) measurements of calcidiol in the serum of healthcare professionals in the midst of the 2021 coronavirus crisis to study the relationship between vitamin D supply and risk of infection in this specific group.

## 2. Results

**Figure 1:**
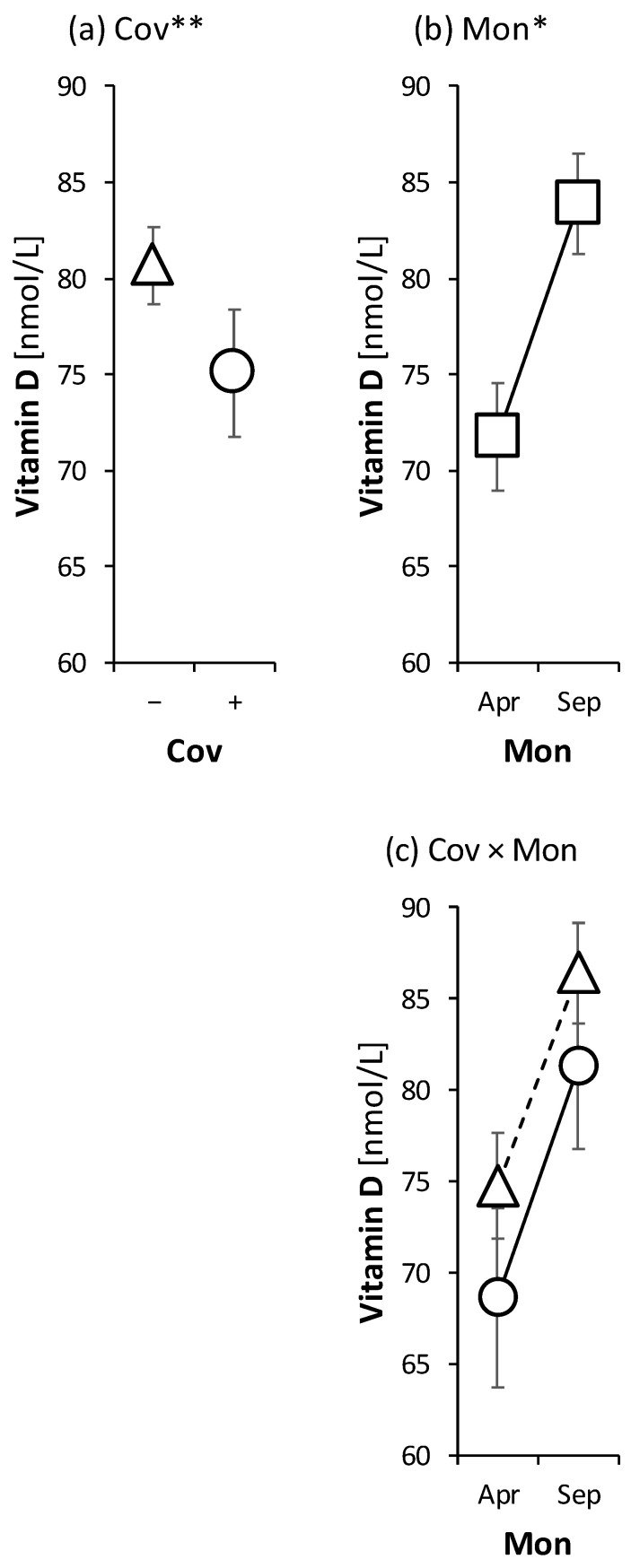
Analysis of relationships between levels of vitamin D and (**a**) the occurrence of COVID-19; (**b**) stage of the trial (month); (**c**) relationships in the interaction of the variables using an ANOVA model. The symbols with error bars represent retransformed means with their 95% confidence intervals. Circles and triangles represent patients with and without occurrence of COVID-19, respectively, (* *p* < 0.05, ** *p* < 0.01). Coronavirus disease 2019, Cov, COVID-19; Mon, month; Analysis of variance, ANOVA.

The relationship between the incidence of COVID-19 disease and the levels of vitamin D measured by LC-MS/MS in men and women of all ages was evaluated using the ANOVA model (see the Statistical Analysis section), and the results are shown in Figure 1.

The first (a) panel of the figure shows an inverse relationship between the occurrence of COVID-19 and the level of vitamin D (factor). The second panel of the figure (b) shows a statistically significant dependence of vitamin D levels on the season. The third panel of the figure (c) shows a comparable effect of the season on the levels of vitamin D in volunteers with and without history of COVID-19 (the interaction between COVID-19 and vitamin D levels was insignificant).

All the subjects who became infected by SARS-CoV-2 had a mild to moderate form of COVID-19 without the need for hospitalization. The mean value of calcidiol in winter was 71 nmol/L, while in summer it was 84 nmol/L. The percentage of probands who confirmed vitamin D supplementation was about 70%. However, none of the participants mentioned the dosage of the substituent, so we could not take this fact into the statistical processing. From the summary of the results, it is clear that it is not the amount of the substituent that matters, but overall vitamin D metabolism and the level of the active form of vitamin D, i.e., calcitriol. The questionnaires showed that only 19% of monitored healthcare staff members did not take vitamin D at all (neither in winter nor in summer).

## 3. Discussion

We have focused our study on clarifying the supportive role of vitamin D in the fight against COVID-19 infection. All the probands were recruited from healthcare professionals and came into daily contact either directly with patients or with biological material that is potentially infectious. We found significantly lower calcidiol levels in participants in the COVID-19 group compared to those without a history of COVID-19 (75.1 vs. 80.70 nmol/L). Our observation is consistent with other studies that highlight the protective effect of vitamin D against COVID-19 when maintaining concentration levels between 75 and 125 nmol/L [18].

Some studies found very low levels of calcidiol and impaired vitamin D metabolism in patients hospitalized with acute COVID-19 [19]. Others have observed a remarkably high percentage (59%) of patients with COVID-19 being vitamin D deficient (calcidiol < 50 nmol/L) on admission, and an association between low admission calcidiol levels and disease stage and mortality, respectively [20,21]. Moreover, severe calcidiol deficiency (<25 nmol/L) showed a trend of longer hospital stays among hospitalized patients with moderate to severe COVID-19 [22]. However, as stated above, not all studies support the hypotheses regarding the relevance of vitamin D in association with COVID-19 [17]. One can speculate that the conflicting results may be caused by the variability of the performed measurements, the inconsistency in study criteria, variable length or dosage of vitamin D substitution, and non-homogenous groups of participants.

In our study, all the subjects were recruited from a healthcare environment and were in daily contact with patients or potentially infectious biological material. At the time of data acquisition, 25% of the participants had a history of COVID-19, but none required intensive care or hospitalization. Some probands admitted that they were taking vitamin D supplements and yet had low levels of it. We discovered that they took 1 drop of liquid cholecalciferol a day, which contained less than 1000 international units (IU). This was not enough at the time of the epidemic, nor is it sufficient for any prevention or even treatment. The recommended usual dose for an adult is 2000–4000 IU/day [23]. The dosage also depends on the person’s body mass index (BMI). In people with a higher BMI, it is necessary to measure their calcidiol levels and adjust the dose to maintain the vitamin D level at 80 nmol/L at least. When using vitamin D as a drug or as a prevention against viral infection, the dose may be three times higher (6000 IU/day) to reach a level of 125 nmol/L. At such a high dosage, it is possible to prevent unwanted calcification caused by activated osteocalcin by the occasional use of fat-soluble vitamin K2. Activated osteocalcin deposits calcium in the bones, whereas non-activated (by K2 action) osteocalcin inhibits calcium absorption by the bones [24].

People over the age of 60 have to take vitamin D throughout the year. The daily dose fluctuates at around 800 IU of cholecalciferol. This is for situations where there is no risk of any serious infections in the general population. The serum concentration of calcidiol depends on the medical condition (e.g., gastrointestinal tract disorders) and the BMI because there are high, mid, and low responders [25,26].

For medication with vitamin D_3_, it is recommended to control the serum level so as to achieve a calcidiol level of at least 80 nmol/L. Calcitriol is essential for many physiological functions [27], including of both innate and adaptive immune functions [28]. During the pandemic, a lesser-known protective effect of vitamin D was documented [29]. Briefly, this concerns the protective mechanism of vitamin D in the renin–angiotensin system. The entry for SARS-CoV-2 infection is often caused by the penetration of the virus through the vascular endothelium, or respiratory epithelium, where the angiotensin-converting enzyme 2 (ACE2) is expressed. This enzyme plays a crucial role in the renin–angiotensin system, balancing vasoconstriction and vasodilatation effects in cardiovascular homeostasis [30]. The protective role of vitamin D against COVID-19-mediated complications lies in the fact that vitamin D can induce the expression of ACE2 and regulate the immune system [18].

Experts also recommend the use of vitamin K2 and magnesium together with vitamin D to avoid the possible side effects of long-term supplementation with vitamin D3 [31], e.g., vascular calcification [32].

## 4. Materials and Methods

### 4.1. Probands

We contacted the staff of our medical facility with a request for voluntary provision of at least 2 mL of serum twice a year: a winter collection by 15 April 2021 and a summer collection by 15 September 2021. Our study was carried out in the middle of the pandemic at a time when each of the probands could come into contact with SARS-CoV-2 infections on a daily basis. The condition was strict compliance with hygiene regulations ordered by the institution’s crisis staff. The volunteers received a patient information form and signed informed consent forms in accordance with the approval of the study proposal by the Ethics Committee of the Institute of Endocrinology on 9 March 2021. In both samples, the probands completed a questionnaire as to whether or not they had contracted COVID-19, whether they were using vitamin D_3_ substitution, and their gender and the date of the sample. The volunteers were rewarded for lost time and inconvenience. The aim of our study was to map vitamin D levels in people who had not had COVID-19 and to compare the supply of vitamin D (calcidiol) in people who had the disease.

### 4.2. Chemicals and Materials

The solvents and chemicals used were obtained from the following suppliers: methanol, CHROMASOLV™ LC-MS Ultra, and water, LC-MS Ultra CHROMASOLV™, from Honeywell Riedel-de Haën™ (Charlotte, NC, USA); acetonitrile gradient grade for LC from Merck KGaA (Darmstadt, Germany); and n-hexane ≥ 99%, HiPerSolv CHROMANORM^®^, for LC-MS and formic acid (FA) eluent additive ≥ 99%, HiPerSolv CHROMANORM^®^, for LC-MS from VWR Chemicals BDH^®^ (Randor, PA, USA).

All the vitamin D standards (25-hydroxyvitamin D2, 25-hydroxyvitamin D3, 3-epi-25-hydroxyvitamin D2, 3-epi-25-hydroxyvitamin D3, 24,25-dihydroxyvitamin D3, and deuterium-labeled d6-25-hydroxyvitamin D3) used as an internal standard (IS) were purchased from Sigma-Aldrich (St. Louis, MO, USA). All the stock solutions were held in methanol and stored in amber vials in the dark at −20 °C to avoid light-induced degradation.

The blood collection system VACUETTE^®^ from Greiner Bio-One International GmbH was used (Kremsmünster, Austria) for sampling.

### 4.3. Instrumentation

The Exion LC AD system was used for the HPLC separation, consisting of an autosampler, a binary pump, a degasser, and a column oven. The MS/MS detection was performed on a Sciex QTRAP 6500 + mass spectrometer (Framingham, MA, USA). The mass spectrometer and the data were operated and processed using Analyst software from Sciex.

### 4.4. Blood Sample Collection, Handling, and Storage

Blood samples were obtained from 56 participants. After collection, the whole blood samples were left undisturbed at room temperature for 30 min. After centrifugation at 1500× g for 10 min in a refrigerated centrifuge, the upper layer was transferred into vials and kept frozen at −20 °C until sample processing.

### 4.5. Sample Processing

Serum samples were thawed at room temperature and vortex-mixed. Then, 200 µL of serum and 10 µL of IS solution were transferred into 2 mL polypropylene microcentrifuge tubes and mixed for 15 s. After adding 200 µL of the acetonitrile/methanol (90:10, *v/v*) mixture and 10 min precipitation, the analytes were extracted with 1 mL of hexane for 1 min. The samples were centrifuged at 15,000× g for 10 min. After centrifugation, 850 µL of supernatant was transferred into clean glass tubes and evaporated under reduced pressure. The dried sample was reconstituted in 75 µL of LC-MS-grade methanol/water (40:60, *v*/*v*), and a 20 µL aliquot of the reconstituted sample was injected into the LC-MS system and analyzed.

### 4.6. LC-MS/MS Conditions

Chromatographic separation was achieved using a Kinetex^®^ 2.6 µm F5 100 Å, LC column 100 × 2.1 mm, from Phenomenex (Torrance, CA, USA) and binary flow gradient with water and methanol as mobile phases. Formic acid was added to a final concentration of 0.1% to both mobile phases as an additive, and the column oven was maintained at 30 °C throughout the analysis. The following gradient was used (Table 1), which enables selective quantification of 25-hyroxyvitamin D3 and its separation from the most common interferents: 25-hydroxyvitamin D2, 3-epimer of 25-hydroxyvitamin D2, 3-epimer of 25-hydroxyvitamin D3, and 24,25-dihydroxyvitamin D3. Deuterated d6-25-hydroxyvitamin D3 was used as an internal standard to improve the precision and accuracy of the determination.

The mass spectrometer was operated using the positive electrospray ionization (ESI) mode. The multiple reaction monitoring (MRM) mode of operation was used with two MRM transitions per analyte (Table 2). Quantification and confirmation MRM transitions were selected based on the optimization of the infusion solution of individual analytes with a concentration of 100 ng/mL.

### 4.7. Method Validation

The method used was tested and validated according to current FDA guidelines [33]. The quality control (QC) samples were prepared using low-level serum and spiked serum samples at four concentration levels covering the calibration range 0.5–100 nmol/L. The QC samples were measured with five replicates and on three different days. The lowest limit of quantification (LLOQ) was 0.5 nmol/L, and the method performance characteristics satisfied the criteria for successful validation. The inter- and intra-assay precisions did not exceed 8%; the accuracy of all measurements ranged from 92 to 107%; and eminent linearity over the whole calibration range was observed with r > 0.999. Moreover, commercial controls with guaranteed values (ClinChek^®^ Serum Control, lyophil., for 25-OH Vitamin D2/D3, Level I, II, from RECIPE (München, Germany)), were used to assure the quality of the performed analyses.

### 4.8. Statistical Evaluation

We used an ANOVA model consisting of a subject factor (explaining the inter-individual variability), between-subject factor occurrence of COVID-19 (+ vs. −), a within-subject factor for month (September vs. April; stage), and a between-factor interaction. For the post hoc analysis, the least significant multiple comparisons were used. To eliminate skewed data distribution and heteroscedasticity, the original data were transformed by a power transformation to attain a symmetric distribution of the dependent variables and, at the same time, to stabilize the variance (attaining homoscedasticity), as described in detail previously [34]. After performing the statistical tests, the data were retransformed into the original scale (for presentation in the figures), using a recurrent formula. The symmetry of the data and residuals and homoscedasticity in the data were checked as previously described [35]. Statgraphics Centurion 18 statistical software from Statgraphics Technologies (The Plains, VA, USA) was used for calculations.

## 5. Conclusions

Several studies recommend a dose of 2000 IU/day. In our opinion, during the epidemic, this should be considered the minimum dose. It is important to measure the level of vitamin D (calcidiol) and increase the dose to reach a minimum level of 80 nmol/L under normal circumstances and to 150 nmol/L during the epidemic.

## Figures and Tables

**Table 1:** Binary flow gradient for the 25-OH vitamin D3 analysis. A flow rate of 0.4 mL/min was maintained throughout the analysis.

Time (min)	%B
0.00	40
0.20	40
0.30	75
4.50	84
4.60	97.5
5.20	97.5
5.25	40
6.10	40
7.00	40

**Table 2:** Summary of retention times (RT) and selected MRM (multiple reaction monitoring) transitions.

Analyte	RT	Quantification Q1/Q3	Confirmation Q1/Q2
25-hydroxyvitamin D3	3.74	383.2/257.2	383.2/365.3
25-hydroxyvitamin D2	3.92	395.1/209.1	395.1/377.1
3-epi-25-hydroxyvitamin D3	3.90	383.2/257.2	383.2/365.3
3-epi-25-hydroxyvitamin D2	4.06	395.1/209.1	395.1/377.1
24,25-dihydroxyvitamin D3	2.41	399.1/381.3	399.1/215.1
d6-25-hydroxyvitamin D3	3.72	389.2/371.2	-

## Data Availability

Not applicable.

## References

[1] Bičíková M., Máčová L., Jandová D., Třískala Z., Hill M. (2021). Movement as a Positive Modulator of Aging. Int. J. Mol. Sci..

[2] Vieth R. (2020). Vitamin D supplementation: Cholecalciferol, calcifediol, and calcitriol. Eur. J. Clin. Nutr..

[3] Xu J., Zhou Y., Yan C., Wang X., Lou J., Luo Y., Gao S., Wang J., Wu L., Gao X. (2021). Neurosteroids: A novel promise for the treatment of stroke and post-stroke complications. J. Neurochem..

[4] BiČíková M., Duskova M., Vítků J., Kalvachová B., Řípová D., Mohr P., Stárka L. (2015). Vitamin D in Anxiety and Affective Disorders. Physiol. Res..

[5] Nithila M.R., Roy N.M., Al-Harthi L., Sampat N., Al-Mujaini R., Mahadevan S., Al Adawi S., Essa M.M., Al Subhi L., Al-Balushi B. (2021). Impact of vitamin D on neurocognitive function in dementia, depression, schizophrenia and ADHD. Front. Biosci..

[6] Carlberg C. (2019). Nutrigenomics of Vitamin D. Nutrients.

[7] Grant W.B. (2019). Review of Recent Advances in Understanding the Role of Vitamin D in Reducing Cancer Risk: Breast, Colorectal, Prostate, and Overall Cancer. Anticancer Res..

[8] Abdi F., Ozgoli G., Rahnemaie F.S. (2019). A systematic review of the role of vitamin D and calcium in premenstrual syndrome. Obstet. Gynecol. Sci..

[9] Ciavattini A., Serri M., Carpini G.D., Morini S., Clemente N. (2016). Ovarian endometriosis and vitamin D serum levels. Gynecol. Endocrinol..

[10] Kalaitzopoulos D.R., Lempesis I.G., Athanasaki F., Schizas D., Samartzis E.P., Kolibianakis E.M., Goulis D.G. (2019). Association between vitamin D and endometriosis: A systematic review. Hormones.

[11] Giampaolino P., Corte L.D., Foreste V., Bifulco G. (2019). Is there a Relationship Between Vitamin D and Endometriosis? An Overview of the Literature. Curr. Pharm. Des..

[12] Ao T., Kikuta J., Ishii M. (2021). The Effects of Vitamin D on Immune System and Inflammatory Diseases. Biomolecules.

[13] Charoenngam N., Holick M.F. (2020). Immunologic Effects of Vitamin D on Human Health and Disease. Nutrients.

[14] Dissanayake H.A., de Silva N.L., Sumanatilleke M., de Silva S.D.N., Gamage K.K.K., Dematapitiya C., Kuruppu D.C., Ranasinghe P., Pathmanathan S., Katulanda P. (2021). Prognostic and Therapeutic Role of Vitamin D in COVID-19: Systematic Review and Meta-analysis. J. Clin. Endocrinol. Metab..

[15] Ghasemian R., Shamshirian A., Heydari K., Malekan M., Alizadeh-Navaei R., Ebrahimzadeh M.A., Warkiani M.E., Jafarpour H., Bazaz S.R., Shahmirzadi A.R. (2021). The role of vitamin D in the age of COVID-19: A systematic review and meta-analysis. Int. J. Clin. Pract..

[16] Pereira M., Dantas Damascena A.D., Galvão Azevedo L.M.G., de Almeida Oliveira T.D.A., da Mota Santana J.D.M. (2022). Vitamin D deficiency aggravates COVID-19: Systematic review and meta-analysis. Crit. Rev. Food Sci. Nutr..

[17] Zelzer S., Prüller F., Curcic P., Sloup Z., Holter M., Herrmann M., Mangge H. (2021). Vitamin D Metabolites and Clinical Outcome in Hospitalized COVID-19 Patients. Nutrients.

[18] Hadizadeh F. (2020). Supplementation with vitamin D in the COVID-19 pandemic?. Nutr. Rev..

[19] Povaliaeva A., Bogdanov V., Pigarova E., Dzeranova L., Katamadze N., Malysheva N., Ioutsi V., Nikankina L., Rozhinskaya L., Mokrysheva N. (2022). Impaired Vitamin D Metabolism in Hospitalized COVID-19 Patients. Pharmaceuticals.

[20] De Smet D., De Smet K., Herroelen P., Gryspeerdt S., A Martens G. (2020). Serum 25(OH)D Level on Hospital Admission Associated With COVID-19 Stage and Mortality. Am. J. Clin. Pathol..

[21] Diaz-Curiel M., Cabello A., Arboiro-Pinel R., Mansur J.L., Heili-Frades S., Mahillo-Fernandez I., Herrero-González A., Andrade-Poveda M. (2021). The relationship between 25(OH) vitamin D levels and COVID-19 onset and disease course in Spanish patients. J. Steroid Biochem. Mol. Biol..

[22] Reis B.Z., Fernandes A.L., Sales L.P., Santos M.D., dos Santos C.C., Pinto A.J., Goessler K.F., Franco A.S., Duran C.S.C., Silva C.B.R. (2021). Influence of vitamin D status on hospital length of stay and prognosis in hospitalized patients with moderate to severe COVID-19: A multicenter prospective cohort study. Am. J. Clin. Nutr..

[23] Grant W.B., Lahore H., McDonnell S.L., Baggerly C.A., French C.B., Aliano J.L., Bhattoa H.P. (2020). Evidence that Vitamin D Supplementation Could Reduce Risk of Influenza and COVID-19 Infections and Deaths. Nutrients.

[24] Yasui T., Miyatani Y., Tomita J., Yamada M., Uemura H., Miura M., Irahara M. (2006). Effect of vitamin K_2_ treatment on carboxylation of osteocalcin in early postmenopausal women. Gynecol. Endocrinol..

[25] Pizzini A., Aichner M., Sahanic S., Böhm A., Egger A., Hoermann G., Kurz K., Widmann G., Bellmann-Weiler R., Weiss G. (2020). Impact of Vitamin D Deficiency on COVID-19—A Prospective Analysis from the CovILD Registry. Nutrients.

[26] Carlberg C., Haq A. (2018). The concept of the personal vitamin D response index. J. Steroid Biochem. Mol. Biol..

[27] DeLuca H.F. (2004). Overview of general physiologic features and functions of vitamin D. Am. J. Clin. Nutr..

[28] Elamir Y.M., Amir H., Lim S., Rana Y.P., Lopez C.G., Feliciano N.V., Omar A., Grist W.P., Via M.A. (2021). A randomized pilot study using calcitriol in hospitalized COVID-19 patients. Bone.

[29] Amraei R., Rahimi N. (2020). COVID-19, Renin-Angiotensin System and Endothelial Dysfunction. Cells.

[30] Shukla A.K., Banerjee M. (2021). Angiotensin-Converting-Enzyme 2 and Renin-Angiotensin System Inhibitors in COVID-19: An Update. High Blood Press. Cardiovasc. Prev..

[31] Goddek S. (2020). Vitamin D3 and K2 and their potential contribution to reducing the COVID-19 mortality rate. Int. J. Infect. Dis..

[32] Myneni V.D., Mezey E. (2016). Regulation of bone remodeling by vitamin K2. Oral Dis..

[33] (2018). Bioanalytical Method Validation Guidance for Industry.

[34] Meloun M., Hill M., Militky J., Kupka K. (2000). Transformation in the PC-Aided Biochemical Data Analysis. Clin. Chem. Lab. Med..

[35] Meloun M., Militký J., Hill M., Brereton R.G. (2002). Crucial problems in regression modelling and their solutions. Analyst.

